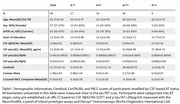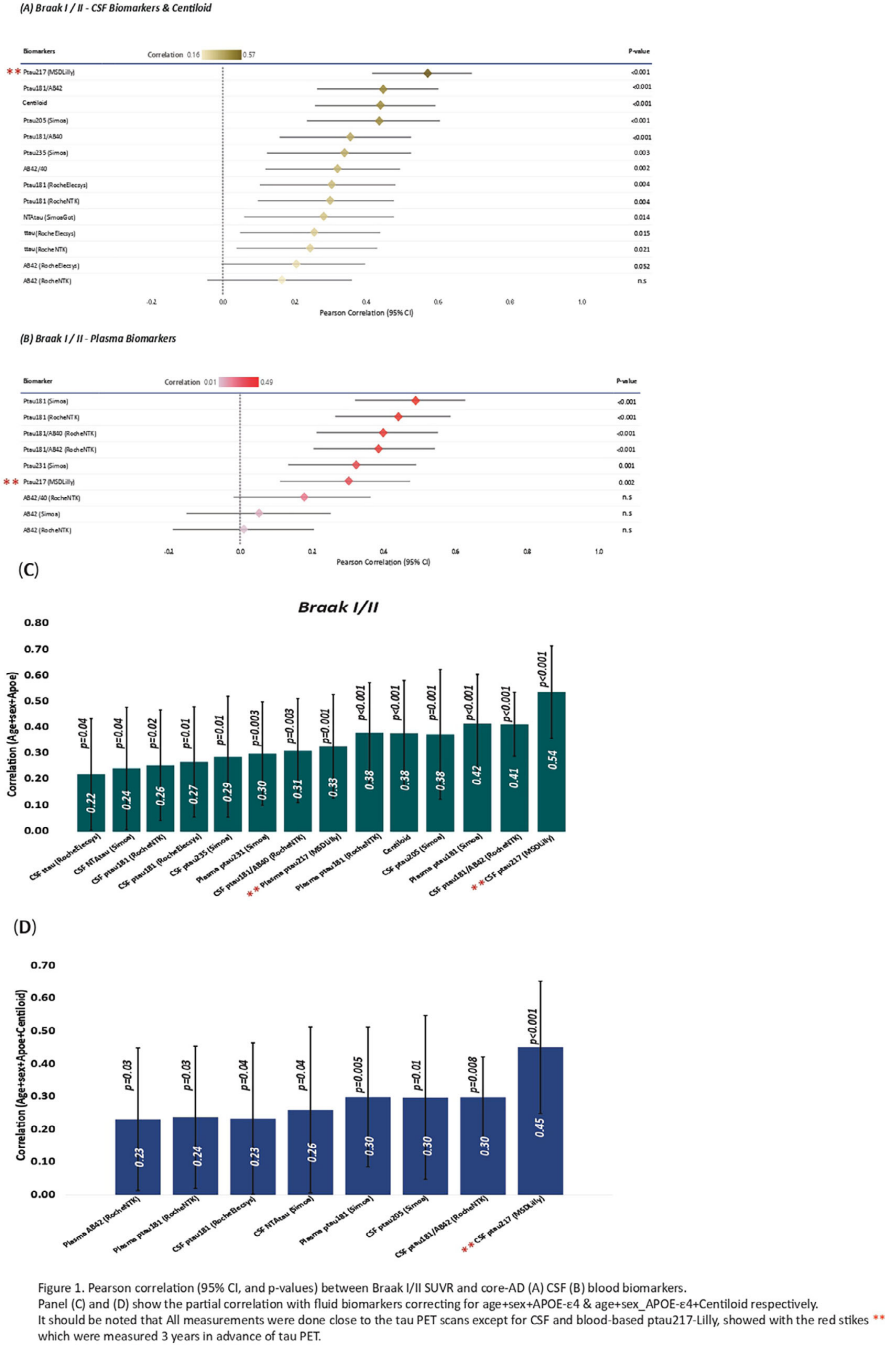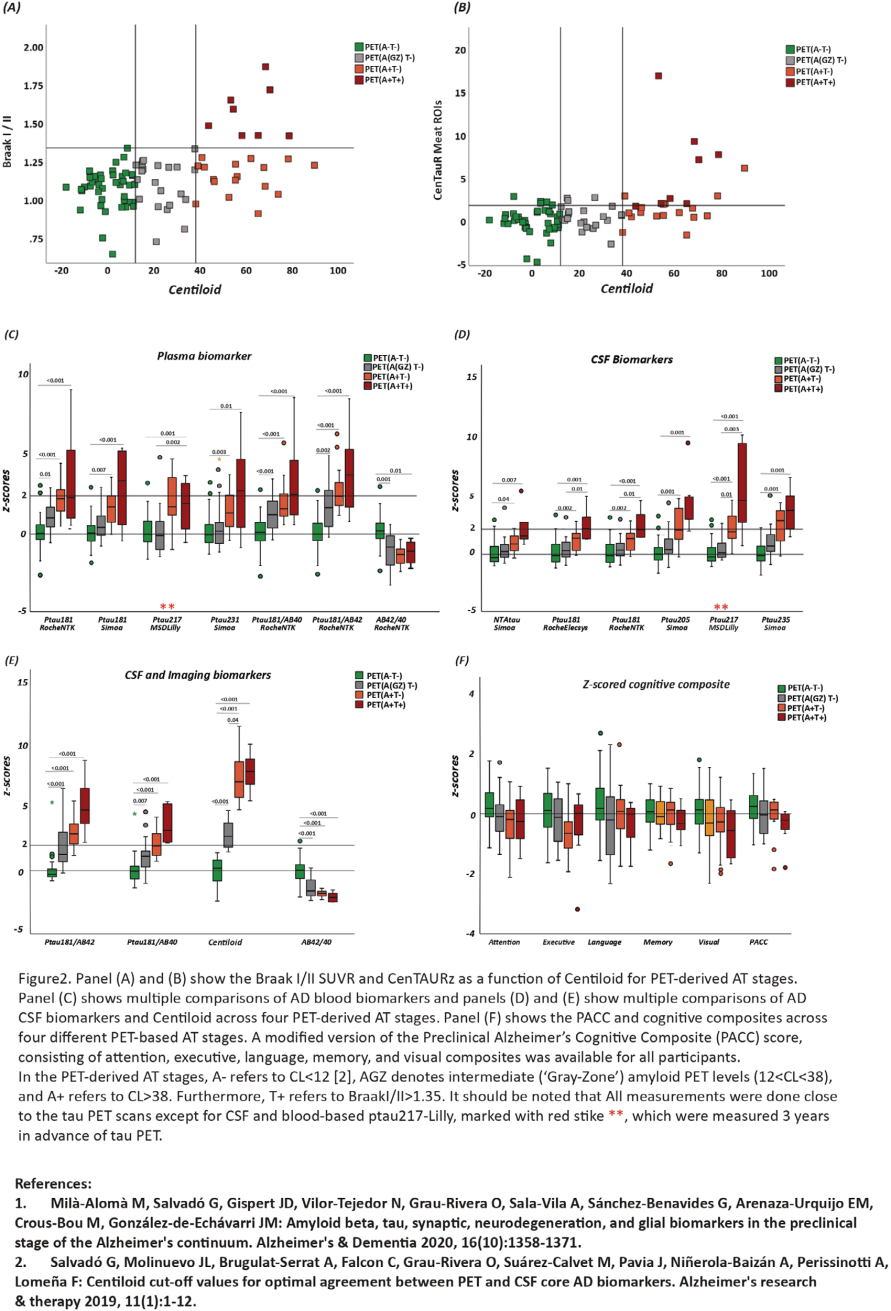# Associations of 18F‐RO‐948 Tau PET with Fluid AD Biomarkers, Centiloid, and Cognition in the Early AD Continuum

**DOI:** 10.1002/alz.093976

**Published:** 2025-01-09

**Authors:** Mahnaz Shekari, Armand González Escalante, Marta Milà‐Alomà, Carles Falcon, David López‐Martos, Gonzalo Sánchez‐Benavides, Anna Brugulat‐Serrat, Aida Niñerola‐Baizán, Nicholas J. Ashton, Thomas K Karikari, Juan Lantero Rodriguez, Anniina Snellman, Theresa A. Day, Jeffrey L. Dage, Paula Ortiz‐Romero, Matteo Tonietto, Edilio Borroni, Gregory Klein, Gwendlyn Kollmorgen, Clara Quijano‐Rubio, Eugeen Vanmechelen, Carolina Minguillón, Karine Fauria, Andrés Perissinotti, Jose Luis Molinuevo, Henrik Zetterberg, Kaj Blennow, Oriol Grau‐Rivera, Marc Suárez‐Calvet, Juan Domingo Gispert

**Affiliations:** ^1^ Barcelona?eta Brain Research Center (BBRC), Pasqual Maragall Foundation, Barcelona Spain; ^2^ IMIM (Hospital del Mar Medical Research Institute), Barcelona Spain; ^3^ Centro de Investigación Biomédica en Red Bioingeniería, Biomateriales y Nanomedicina (CIBER‐BBN), Instituto de Salud Carlos III, Madrid Spain; ^4^ Nuclear Medicine Department, Hospital Clínic Barcelona, Barcelona Spain; ^5^ NIHR Biomedical Research Centre for Mental Health & Biomedical Research Unit for Dementia at South London & Maudsley NHS Foundation, London United Kingdom; ^6^ Department of Psychiatry and Neurochemistry, Institute of Neuroscience and Physiology, The Sahlgrenska Academy, University of Gothenburg, Mölndal, Gothenburg Sweden; ^7^ Turku PET Centre, University of Turku, Turku Finland; ^8^ Lilly Research Laboratories, Eli Lilly and Company, Indianapolis, IN USA; ^9^ Roche Pharma Research and Early Development, Neuroscience and Rare Diseases Biomarkers, F. Hoffmann‐La Roche Ltd., Basel Switzerland; ^10^ Roche Pharma Research and Early Development, FHoffmann‐La RocheLtd, Basel Switzerland; ^11^ Roche Pharma Research and Early Development, Neuroscience and Rare Diseases Biomarkers, Basel Switzerland; ^12^ Roche Diagnostics GmbH, Penzberg Germany; ^13^ Roche Diagnostics International Ltd., Rotkreuz Switzerland; ^14^ ADx NeuroSciences NV, Technologiepark 94, Gent Belgium; ^15^ Barcelona?eta Brain Research Center (BBRC), Barcelona Spain; ^16^ Centro de Investigación Biomédica en Red de Bioingeniería, Biomateriales y Nanomedicina (CIBER‐BBN), Madrid Spain; ^17^ Lundbeck A/S, Copenhagen Denmark; ^18^ Department of Neurodegenerative Disease, UCL Institute of Neurology, London United Kingdom; ^19^ Clinical Neurochemistry Laboratory, Sahlgrenska University Hospital, Mölndal Sweden; ^20^ Centro de Investigación Biomédica en Red Bioingeniería, Biomateriales y Nanomedicina, Instituto de Salud Carlos III, Madrid Spain

## Abstract

**Background:**

Fluid biomarkers provide a convenient way to predict AD pathophysiology. However, few studies have focused on determining associations with tau neurofibrillary tangle pathology in the early preclinical AD continuum, relevant to prevention strategies.

**Methods:**

Ninety‐nine cognitively unimpaired individuals from the ALFA+ cohort with valid 18F‐RO‐948 and 18F‐flutemetamol PET, T1‐weighted MRI, cognition, CSF, and plasma biomarkers were included. Participants were initially categorized into AT stages using CSF‐based pre‐established cut‐off values [1]. Regional SUVR of 18F‐RO‐948 PET was calculated in entorhinal (BraakI/II), limbic (BraakIII/IV), and neocortical (BraakV/VI) regions using the inferior cerebellum as reference region as well as with the CenTAURz. Regional positivity thresholds per Braak stage were calculated as the median+2SD of the CSF A‐T‐ group. Amyloid PET was quantified using Centiloids. Pearson correlations were calculated between regional 18F‐RO‐948 SUVRs and AD biomarkers. ROC analyses adjusted for age, sex, and APOE‐e4 performed to evaluate the capacity of biomarkers in predicting BraakI/IIPositive. Four progressive PET‐derived AT groups were defined using Centiloid and tau PET positivity cut‐offs (A‐T‐, AGZT‐, A+T‐ and A+T+; with A‐ CL<12, 12=AGZ<38 and A+ CL=38 [2], and T+ BraakI/II>1.35) and between‐stage differences in z‐scored biomarkers evaluated using a Kruskal‐Wallis tests.

**Results:**

Table 1 shows demographic information of participants. Nine(9.09%) participants were BraakI/IIPositive, seven(7.07%) BraakIII/IVPositive and one(1.01%) BraakV/VIPositive. Two BraakIII/IVPositive participants were BraakI/IINegative, deviating from the Braak hierarchical model. CSF biomarker correlations with BraakI/II SUVR (Figure 1‐A) ranged from r=0.24(ttau) to r=0.57(ptau217) and plasma (Figure 1‐B) from r=0.30(ptau217) to r=0.49(ptau181). Correlations survived adding age+sex+APOE‐e4 in the model (Figure 1‐C&D). CSF ptau181/Aß42, ptau217 and ptau205 showed an AUC=0.93 to predict BraakI/IIPositive, and plasma ptau181, ptau181/Aß42 and ptau217 had an AUC=0.84. Centiloid positivity threshold for BraakI/IIPositive was 38.14CL. Plasma ptau181, ptau181/Aß42, and CSF ptau205, ptau217, and ptau235 reached a mean z‐score>2 for the PET‐derived A+T+ group (Figure 2) which was associated with lower cognitive scores for executive function (p=0.03), attention (p=0.05), and the PACC (p=0.01).

**Conclusion:**

18F‐RO‐948 PET conformed to the Braak hierarchical model for most tau‐positive participants. Fluid AD biomarkers showed moderate associations with tau PET SUVR. Plasma biomarkers showed good capacity to predict BraakI/IIPositive and track fibrillary amyloid and tau pathological changes in the early preclinical AD continuum.